# Is It Time to Reconsider Rituximab Dosing Regimens for Pemphigus Vulgaris?

**DOI:** 10.3390/antib13010004

**Published:** 2024-01-05

**Authors:** Christian Ciolfi, Jacopo Tartaglia, Mauro Alaibac

**Affiliations:** Dermatology Unit, Department of Medicine (DIMED), University of Padua, 35121 Padua, Italy; chriciolfi96@gmail.com (C.C.); jacopo.tartaglia@studenti.unipd.it (J.T.)

**Keywords:** pemphigus vulgaris, rituximab, very ultra-low dose, infusion reactions, cost-effectiveness

## Abstract

Rituximab is currently approved for patients affected by moderate-to-severe pemphigus vulgaris, a severe autoimmune blistering skin disease that can be life-threatening. The standard rituximab dosing regimens, originally established for B-cell non-Hodgkin’s lymphomas, have been recognized to exceed the effective dose required for inducing B-cell depletion, considering that the B-cell burden in pemphigus vulgaris is considerably lower than in lymphoproliferative disorders. We herein report our experience with very ultra-low-dose rituximab in two patients affected by pemphigus vulgaris.

## 1. Introduction

Pemphigus refers to a group of autoimmune blistering dermatoses affecting the skin and the mucous membranes, characterized by intraepidermal acantholysis and consequent formation of blisters and erosions. Different clinical subtypes are recognized, including pemphigus vulgaris (PV), pemphigus foliaceus, IgA pemphigus, pemphigus herpetiformis, and paraneoplastic pemphigus, with PV being the most common subtype. Regardless of pemphigus subtypes, intraepidermal acantholysis is due to circulating autoantibodies targeting desmogleins 1 (DSG1) and 3 (DSG3), which are transmembrane glycoproteins of desmosomes contributing to cell-to-cell adhesion between keratinocytes [1]. Conventional treatments for PV include high doses of systemic corticosteroids and adjuvant steroid-sparing immunosuppressor and/or immunomodulant approaches such as azathioprine, mycophenolate mofetil, methotrexate, dapsone, tetracyclines, plasmapheresis, and high-dose intravenous immunoglobulins [2]. In 2018, rituximab (RTX) was approved by the FDA for adult patients with moderate-to-severe PV in combination with corticosteroids. RTX is a chimeric murine/human monoclonal antibody targeting CD20, a transmembrane surface molecule expressed as homo-dimers or homo-tetramers by pre-B lymphocytes and B lymphocytes, but not by pre-B hematopoietic stem cells and terminally differentiated plasma cells [3]. RTX acts mainly through antibody-dependent cell-mediated cytotoxicity, although other mechanisms may be involved, including antibody-dependent cellular phagocytosis, direct apoptosis induction, and complement-dependent cytotoxicity [4]. In patients with PV, RTX acts through B cells and lymphoid resident memory B-cell depletion, with consequent decrease in circulating pathogenetic anti-DSG autoantibodies; however, it also seems to deeply modulate immune function in PV, as suggested by disease remission frequently lasting longer than B-cell reconstitution in the peripheral blood. In particular, RTX indirectly downregulates autoreactive CD4+ Th cells by depleting B lymphocytes serving as antigen-presenting cells [3,5].

The optimal RTX dosing regimen to adopt in PV is still a matter of debate. Conventional RTX dosing regimens for PV, initially adapted from the protocols used in lymphoproliferative diseases, may exceed the required doses for inducing B-cell depletion. Recent studies support low-dose and ultra-low-dose RTX regimens for PV treatment [6,7].

Recently, we have successfully treated two patients suffering from PV with very ultra-low-dose RTX (a single intravenous infusion of 20 mg). In this case series, we present a comprehensive overview of these patients’ clinical profiles and of the treatment protocol employed.

## 2. Case Presentation

Patient 1 was a 30-year-old woman with a 5-year history of PV affecting the oral cavity. Over the years she had received multiple therapies, including high-dose systemic corticosteroids, azathioprine, and RTX (two infusions of 1000 mg RTX at a 2-week interval were scheduled). However, during the first RTX infusion, she experienced tongue swelling with associated shortness of breath, which was interpreted as an allergic reaction. Consequently, further RTX infusions were contraindicated, and the patient was transitioned to treatment with rilzabrutinib (as part of the phase 3 randomized, parallel-group, double-blind, placebo-controlled trial PEGASUS of oral rilzabrutinib PRN1008 vs. placebo in moderate-to-severe PV). The patient experienced significant benefits from rilzabrutinib therapy, achieving complete remission of disease; however, rilzabrutinib treatment was discontinued due to trial termination. Several months later, the patient came to our attention with a recurrence of oral cavity PV. On examination, painful erosions were evident, affecting all areas of the oral cavity, with notable involvement of the retro-molar trigone and the buccal mucosa; concurrent desquamative gingivitis was also present. Remarkably, there were no lesions observed on the lips or cutaneous surfaces throughout the course of the disease. The patient reported difficulties in eating and swallowing and severe pain. Enzyme-linked immunosorbent assay identified serum antibodies against DSG 1 and DSG 3: 131.62 U/mL and 76.18 U/mL, respectively. In order to minimize the risk of hypersensitivity reactions, we decided to treat the patient with a single infusion of 20 mg RTX with premedication with chlorphenamine maleate 10 mg, paracetamol 1 g, and methylprednisolone 20 mg. The patient also received a short course of oral corticosteroids (starting with prednisone 25 mg/day for 10 days, then gradually tapered over a few weeks). The infusion was well tolerated, and the patient showed significant clinical improvement within a few weeks.

Patient 2 was a 58-year-old woman with a 2-year history of PV affecting the oral cavity and the trunk. Over the years, she had been treated with high-dose systemic corticosteroids and azathioprine; in June 2022, she received a single infusion of 200 mg RTX (ultra-low dose), which had been stopped due to the development of a tingling sensation in the tongue and throat. On examination, widespread erosions and crusty plaques were observed on the back, presternal region, abdomen, upper and lower limbs, and scalp; the patient also reported difficulties in eating and swallowing due to painful erosions involving gingival, buccal, and palatal mucosa. Enzyme-linked immunosorbent assay identified serum antibodies against DSG1 and DSG3: 89.2 U/mL and 142.11 U/mL, respectively. In order to minimize the risk of hypersensitivity reactions, we treated the patient with a single infusion of 20 mg RTX with premedication with chlorphenamine maleate 10 mg, paracetamol 1 g, and methylprednisolone 20 mg. The patient also received a course of oral corticosteroids (starting with prednisone 50 mg/day for 14 days, then gradually tapered over a few weeks). The infusion was well tolerated, and the patient improved significantly within a few weeks.

Peripheral blood B cells were measured before and two weeks after the infusion: at baseline, the patients had B cells within the normal range; post-treatment, CD19/20+ cells dropped to 0% in both patients. Follow-up evaluations at 4 months showed sustained clinical remission without any noteworthy adverse effects for both patients.

## 3. Discussion

There is still no consensus on the RTX dosing regimen best suited for PV. At first, patients had been treated with 4 weekly infusions of 375 mg/m^2^ RTX, according to lymphoma protocols. Nowadays, the American and European guidelines recommend an induction regimen consisting of two infusions of 1000 mg RTX at a 2-week interval with a tapering course of glucocorticoids [2]. Regarding the maintenance regimen, American guidelines propose 500 mg RTX infusion at month 12, with subsequent infusions every six months based on patients’ clinical conditions [8], while European guidelines suggest 500 mg RTX infusions at months 12 and 18, followed by subsequent infusions every six months based on patients’ clinical conditions [9]. However, it should be noted that in autoimmune blistering skin diseases like PV, the B-cell burden is considerably lower than in lymphoproliferative diseases, therefore standard approved RTX dosages may exceed the effective dose required for inducing B-cell depletion. A first interesting starting point came from the open-label, exploratory trial conducted by Schoergenhofer C. and co-authors, who demonstrated that in healthy volunteers, <1% of the conventional RTX doses was enough to deplete circulating B lymphocytes (doses of 1 mg/m^2^, 0.3 mg/m^2^, and 0.1 mg/m^2^ RTX depleted 97%, 75%, and 66% of peripheral B cells, respectively) [6]. Furthermore, while standard RTX regimens have shown promising results in achieving clinical remission, they also pose concerns related to potential side effects and high costs. All these concerns have prompted clinicians to explore alternative dosing protocols. The authors of the present article recently reviewed lower-dose regimens of RTX for the treatment of PV, highlighting how the existing literature supports the use of low-dose and ultra-low-dose RTX protocols to treat PV, with the possibility of repeated infusions for more severe cases [7]. It is important to highlight that dose-defining trials for PV are missing. The systematic review and meta-analysis by Wang HH and co-authors examined different RTX treatment regimens for PV. High-dose RTX (≥2000 mg/cycle) did not show significant superiority over low-dose protocols (<1500 mg/cycle) in terms of complete remission, time to control, and relapse rates. However, high-dose RTX led to longer complete remission maintenance [10]. More specifically, in a study on 27 subjects, high-dose RTX reduced the time to complete remission and relapse rates [11], while in a trial involving 22 PV patients, there were no significant differences in terms of complete remission and time to control [12]. Another study involving 23 patients found similar outcomes between high and low doses, particularly in severe PV cases [13]. In a recent pilot study involving eight PV patients receiving a single 200 mg RTX infusion, five subjects achieved complete remission while three subjects showed a partial response. At the end of the observation period, one patient was no longer under systemic corticosteroids, while another one experienced a relapse [14].

Our experience provides insights into the potential of very ultra-low-dose RTX as an effective and safe option for patients with PV. Although further larger clinical trials are certainly needed to confirm our hypothesis, we support lower RTX dosages as a promising strategy to improve cost-effectiveness and, hopefully, to minimize the risk for infections and adverse events, particularly infusion-related hypersensitivity reactions.

## Data Availability

Data are contained within the article.
